# Active surveillance study of adverse events following immunisation of children in the Czech Republic

**DOI:** 10.1186/s12889-017-4083-4

**Published:** 2017-02-06

**Authors:** Jana Danova, Aneta Kocourkova, Alexander M. Celko

**Affiliations:** Department of Epidemiology, Third Faculty of Medicine Charles University, Prague, Czech Republic

**Keywords:** Adverse events following immunisation, Vaccination, Vaccine, Children

## Abstract

**Background:**

Despite the undisputed public health benefits of routine vaccination, adverse events following immunisation (AEFI) remain a concern. As most adverse events are mild, they may be under-reported; this may underlie the wide range of AEFI rates reported in the literature. We investigated the rates of AEFI related to routine vaccination of children 0–10 years old in the Czech Republic.

**Methods:**

The study reviewed patients’ records in a sample of 49 paediatric GP practices covering all 12 administrative regions of the Czech Republic between 2011 and 2013. Adverse events following routine immunisation of children aged 0–10 years were identified and recorded.

**Results:**

The overall rate of AEFI was 209/100,000 doses; this was 6 times higher than the rate reported to the Czech State Institute for Drug Control (34/100,000 doses). Over two fifths (44%) of all AEFI occurred after the booster dose of the combined diphteria, tetanus and pertussis vaccine in 5-year old children. The vast majority of AEFI were non-serious local events (e.g. redness) and fever. Most AEFI occurred the second day after the immunisation, lasted 4 days on average, and were treated by cold therapy, antipyretics and analgesics.

**Conclusions:**

The rate of AEFI identified in this study was considerably higher than the officially reported rate. Although the vast majority of AEFI were non-serious, health care providers and the public should be educated and encouraged to report AEFI to address the issue of underreporting, to increase the safety profile of vaccines, and to improve public confidence in immunisation programmes.

## Background

With over 80% of children worldwide immunized, it has been estimated that vaccines save some 2–3,000,000 million deaths each year [1]. In the Czech Republic, with population of 10 million, vaccination prevents about 500 child deaths and some 150,000 non-fatal episodes of infectious diseases annually [2]. Despite these striking public health benefits, a part of the general public continues raising concerns about mass vaccination. The main criticism relates to the potential vaccine-associated risks, including AEFI, although these events are extremely rare and must be weighed against the protective benefits of vaccines [3, 4].

Adverse events following immunisation can be local (e.g. erythema, oedema, pain) or systemic (e.g. fever, exanthema, allergic reactions), and acute (within minutes of administration) or delayed (several hours or days after administration). Depending on the clinical relevance and severity, AEFI can be classified as physiological and non-physiological [5, 6]. Physiological adverse events, reflecting natural reaction to the vaccine antigen, are common; they often include elevated body temperature, exanthema and myalgia, and usually have short duration [7–9]. Since physiological reactions are believed to be natural, they are rarely reported. Non-physiological AEFI, sometimes referred to as hyper-reactions, are rare, unexpected and more severe than physiological AEFI, and they tend to occur in immunocompromised patients or patients allergic to vaccine components [10, 11]. The most severe AEFI are either allergic (anaphylaxis) or neurological (encephalopathy, encephalitis, neuritis), and can lead to hospitalization or death [12, 13].

AEFI are identified by either active or passive surveillance [14–16]. Active surveillance often uses electronic system for monitoring of adverse events. Active surveillance will detect more AEFI, but the majority will have milder symptoms [17, 18]. Passive surveillance system relies on voluntary reporting of adverse events sent by physicians and patients [19, 20]; such data are often the basis of administrative reports to national institutions. Passive surveillance system has multiple limitations, including unconfirmed diagnoses, under-reporting of less severe adverse events data, and lack of clarity about the temporal link between AEFI and vaccination and the fact that delayed adverse events are less likely to be reported [21]. In addition, population-based active surveillance allows comparisons of rates of AEFI by vaccination status or by temporal period; this is not possible with passive surveillance. Differences in the methods used to identify AEFI are reflected by the wide range of vaccine-induced adverse events (between 4.8 and 83.0 per 100,000 doses) reported in the literature [3, 8], although the frequency of true allergic AEFI to routine vaccination is estimated be around 1 or 2 per 1,000,000 [7, 17]. Given the uncertainty about the frequency of AEFI, particularly the less severe, it is important to collect data directly from physicians who conduct immunisation and who are the most likely point of first contact when AEFI occur [14, 22].

The aim of the presented study was to investigate the frequency and characteristics of AEFI after routine vaccination of children in the Czech Republic, a country with a long tradition of well organised vaccination system and high immunisation coverage. We actively collected data on AEFI in a sample of paediatrician GPs, and compared the findings with the passive surveillance data reported to the State Institute for Drug Control. In the Czech system of collecting data of AEFI uses only passive reporting system from physicians.

## Methods

We conducted a study of paediatric GP records covering the period 1 January 2011 to 31 December 2013. In the Czech Republic, there are separate GP practices for adults and for children and adolescents (ages 0 to 19 years); these practices provide care for children and adolescents, including vaccination. Vaccines included in the routine compulsory immunisation schedule of children in the Czech Republic are shown in Table 1 [8]. Non-physiologic adverse events following immunisation should be reported to the State Institute for Drug Control and the cause of these adverse events should be investigated.

**Table 1:** Vaccines used for routine obligatory vaccination of children in the Czech Republic

DTaP	Diphtheria, tetanus and pertussis vaccine
DTaP-IPV	Diphtheria, tetanus, pertussis and poliomyelitis vaccine
DTaP- Hib- HBV - IPV	Diphtheria, tetanus, pertussis, infections caused by *Haemophilus influenzae* b, viral hepatitis B and poliomyelitis vaccine
MMR	Measles, mumps and rubella vaccine

Physician in the Czech Republic get information from hospitalisations; given the retrospective nature of our study, the relatively short delay should not affect our data.

For this study, a sample of paediatric GPs was randomly selected from a GP practice register. From the 72 invited GPs in each of the 12 regions of the Czech Republic (covering both regional capital cities and settlements outside of the capital city), 49 (68%) agreed to participate. A questionnaire was developed in collaboration with an academic paediatrician, a hospital paediatrician and paediatric GP. All participating paediatric GPs were personally visited in early 2013 and introduced in the study protocol. After approximately 6 months, GPs were visited again and GP records for years 2011 and 2012 were reviewed and AEFI were recorded. Records covering 2013 were reviewed and data collected during a second visit in early 2014. Records of routine obligatory vaccinations of children (0–10 years) were reviewed, and all AEFI were recorded. Our comparison with the national system is therefore based on all AEFI, rather than on the distinction between physiological and non-physiological AEFI. Data recorded for the study included the type of vaccine, characteristics of the adverse events, description, frequency, duration and treatment, and demographic data (age, sex, birthweight, education of parents, place of residence). Paediatric GPs also provided the number of patients registered in their practice and the numbers of doses of routine vaccines they administered in order to estimate the rate of AEFI. The types of AEFI collected in this study are described in Table 3 [9, 10].

## Results

During the study period, 175 AEFI after routine compulsory vaccination of children (age 0–10 years) were identified in the GP records. The overall rate of AEFI after routine vaccination was 209 per 100,000 vaccine doses (Table 2). This is much higher than the official rate of 34 per 100,000 registered in the State Institute for Drug Control during the same period [9]. The highest rates was seen for DTaP vaccine (620/100,000 doses).

**Table 2:** Numbers and rates of AEFI of children identified in paediatric GP records

Vaccine	Number of doses	Number of adverse events	Rate per 100,000 doses
DTaP-Hib-HBV-IPV	36020	55	152.7
MMR	24660	21	85.2
DTaP	12251	76	620.4
DTaP-IPV	10620	23	216.6
Total	83551	175	209.4

Local reactions accounted for 65% of all reported adverse events (Table 3). Redness, swelling and pain often occurred in combination; taken separately, redness was observed in 46%, swelling in 38% and pain in 26% of all reported reactions. Fever occurred in 27% of recorded AEFI. By contrast, serious adverse events, such as apnoea, convulsions, hypotonic- hyporesponsive episodes, encephalopathy were rare, accounting for 0.3, 1.4, 1.4 and 2.2% of all identified adverse events, respectively. More than half of all AEFI (53%) occurred after booster dose of vaccination, most frequently after the revaccination with DTaP vaccine at 5 years of age, followed by the first (26%), second (16%) and third (6%) dose of the DTaP- Hib- HBV-IPV vaccine and first dose of MMR vaccine.

**Table 3:** Frequency, outset and duration of different types of AEFI of children

Type of adverse events following immunisation	Frequency^a^	Outset of symptoms (days after vaccination, mean -range in days)	Duration of symptoms (mean in days)
Local (rednes, swelling, pain)	62.8%	1.6 (1–8)	5.2
Abscess in application site	1.1%	5.5 (2–10)	16.0
Lymphadenitis	1.7%	20.9 (7–31)	44.3
Fever	26.8%	1.3 (1–7)	2.8
Alergic reaction	0.6%	0 (0)	1.5
Posvaccinal exanthema	4.5%	3.3 (2–9)	4.2
Arthralgia	1.7%	1.2 (1–5)	13.2
Excessive crying	4.2%	0.9 (0–4)	1.8
Hypotonic hyperactive epizode	1.4%	0 (0–1)	0.3
Gastrointestinal disorders	5.0%	1.4 (0–5)	2.2
Encephalopathy	2.2%	1.1 (1–7)	4.0
Convulsions	1.4%	0.5 (0–1)	0.5
Apnoe	0.3%	0.5 (0–1)	1.0
Others	8.4%	1.3 (0–8)	7.9

^a^observed more than one reaction per one child

The mean onset of adverse events is shown in Table 3. Most AEFI appeared 1 day after vaccination, some of these events occurred on the same day of vaccination and 2 days after vaccination. However, lymphadenitis as systemic adverse event was seen on average 30 days after vaccination (3 cases), abscess happened 5 days after vaccination (2 cases) and post-vaccination exanthema occurred on average 3 days after vaccination (8 cases). The mean duration of local reaction was 5 days, while fever took about 3 days to resolve.

From 175 recorded AEFI, medical assistance was needed in 28 (16%) instances. Over two fifths (42%) of adverse events required treatment; mostly cold therapy (40%), antipyretics and analgesics (24%), local therapy (17%), antihistamines (13%) and antibiotics (3%). In our study sex, low birth weight, parents education and place of residence were not statistical significant associated with higher frequency of AEFI (Table 4.). The only one statistical significant parameter observed in the study was age of children. Age group 5–9 years was associated with highest frequency of AEFI after application of DTaP vaccine (Table 4.)

**Table 4:** Frequency of AEFI by socio-demograpic variables

	Number of doses	Number of AEFI	Rate (95CI) per 1000 doses	Relative risk (95%CI)	*p*-value
Age group					
0–11 months	27555	41	1.5 (1.1–2.0)	1 .00	<0.001*
1–4 years	33125	35	1.1 (0.7–1.5)	0.71 (0.45–1.12)	
5–9 years	12251	76	6.2 (4.9–7.7)	4.16 (2.81–6.26)	
10–14 years	10620	23	2.2 (1.4–3.2)	1.45 (0.83–2.49)	
Sex					
Boys	42611	94	2.2 (1.8–2.7)	1.05 (0.92–1.21)	0.515
Girls	40940	81	2.0 (1.6–2.5)	1.00	
Birth weight					
< 2500 g	3350	7	2.1 (0.9–4.1)	0.48–2.06)	0.497
> 2500 g	80201	168	2.1 (1.8–2.4)	1.00	
Education of mother					
Elementary	10970	19	1.7 (1.1–2.7)	1.00	0.495*
Secondary	46332	99	2.1 (1.8–2.6)	1.22 (0.75–2.14)	
University	22470	48	2.1 (1.6–2.8)	1.22 (0.71–2.22)	
Not available	3779	9	2.4 (1.2–4.4)	1.38 (0.55–3.19)	
Education of father					
Elementary	14277	20	1.4 ((0.9–2.1)	1.00	0.580*
Secondary	41618	105	2.5 (2.1–3.0)	1.80 (1.11–3.07)	
University	21242	39	1.8 (1.3–2.5)	1.31 (0.75–2.37)	
Not available	6414	15	2.3 (1.4–3.9)	1.67 (0.79–3.43)	
Place of residence					
Rural	18214	36	2.0 (1.4–2.7)	1.00	0.347
Urban	65337	139	2.1 (1.8–2.5)	1.02 (0.94–1.10)	

*p-value for trend

## Discussion

In this study, we actively collected data on adverse events in a sample of paediatric GPs practices. The overall rate of AEFI after routine obligatory vaccination in children identified in our study was 209 per 100,000 vaccine doses, which is 6 times higher than the officially reported rate to the State Institute for Drug Control (an agency officially responsible for recording such events). The vast majority of AEFI were mild and local; the most common systemic AEFI was fever; and only 16% required medical treatment.

Several limitations of this study need to be considered when interpreting these results. First, although the practitioners were selected randomly, some 30% of invited GPs did not participate in the study. However, it is unlikely that non-participating practitioners had very different AEFI rates than those who participated in the study; the non-response therefore should not affect the results.

The second potential limitation is that fact the data on AEFI are based on paediatric GP records, rather than on self-report by the parents of children. It is likely that parents would report more adverse events than paediatricians; on the other hand, physicians are the only persons who are qualified to recognise the adverse event as a non-physiological [19, 20].

The third, and related, limitation is the question of what constitutes a non-physiological AEFI. Although national and WHO criteria exist, the distinction between physiological and non-physiological adverse event is blurred, and the classification may depend on a number of factors, including GPs perceptions and the attitude of parents. The literature on AEFI rates is relatively sparse. Fritsche et al., using data from the US, the Netherlands and Australia, reported a very wide range of AEFI in these countries, between 4.8 and 83.0 per 100,000 doses of vaccines [3]. Surveillance of AEFI in Zhejiang province in China in 2008–2011 found 85 adverse events per 100,000 infants under 1 year of age [8]. As the data in these studies were collected using different methodology in each country, they cannot be directly compared; however, they do indicate potential under-reporting of AEFI [3].

The rate of AEFI in the present study (209 per 100,000 doses) was significantly higher than the rate reported to the State Institute for Drug Control (34 per100,000 doses), reflecting the difference between active surveillance used in our study and passive surveillance relying on reports to the national authority. On the other hand, the reports to the Czech State Institute for Drug Control included much higher proportion of serious AEFI than we found in our data. In our study were included all adverse events that were available in paediatric GP records. We did not exclude any event. There were no AEFI described as purpura or GBS. In 2011, the Czech State Institute for Drug Control received a total of 817 AEFI reports, from which 51% were considered serious [9], compared with 3% serious AEFI in our study. Again, this is likely to reflect differences in data collection. In the study we were focussed to collection of all AEFI described in GP offices.

The Vaccine Adverse Event Reporting System (VAERS), maintained by the US Centre for Disease Control and Prevention and Food and Drug Administration, reported the rate of serious AEFI in 2006–2010 as 8%; this is not too far from the rate seen in our study. Non-serious AEFI are much more common; consistently with our study, local events (exanthema) were the most common symptoms reported after immunisation in an analysis of the Chinese reporting system in 2009 [19].

Training of health care providers and education of the general public may improve the reporting of vaccine safety issues and gradually reduce the mistrust regarding vaccines safety harboured by some segments of the public [20]. In the Czech Republic, there are no detailed guidelines how AEFI should be reported, apart from the newsletter provided by the State Institute for Drug Control [9]. Vaccination registers, which currently exist in in 11 EU countries [20, 23] may be a possible solution but at present there are no plans to establish an immunisation registry in the Czech Republic. Although the vast majority of AEFI identified in this study were mild. An establishment of a vaccination register, which would also collect data on AEFI, should be considered to improve the evidence on this important public health issue. The best way forward would be to have a vaccination registry with linkage available to other data at GPs or hospitals to allow robust epidemiological studies to be done on vaccine safety signals (as WHO blueprint indicates) [http://www.who.int/topics/immunization/en/]. In addition active surveillance of rare but serious events of interest (such as done by rapid cycle analysis in the Vaccine Safety Datalink) would be useful.

## Conclusions

The rate of AEFI identified in this study was considerably higher than the officially reported rate, although the vast majority of AEFI were non-serious and only 16% required medical attention. Nevertheless, given the public concerns about risks associated with immunisation, both health care providers and the general public should be educated about AEFI and health providers should be encouraged to report AEFI to address the issue of underreporting, to increase the safety profile of vaccines, and to improve public confidence in immunisation programmes.

## Abbreviations

AEFI: Adverse events following immunisation
DTaP- Hib-HBV-IPV: Diphtheria, tetanus, pertussis, infections caused by *Haemophilus influenzae* b, viral hepatitis B and poliomyelitis vaccine
DTaP: Diphtheria, tetanus and pertussis vaccine
DTaP-IPV: Diphtheria, tetanus, pertussis and poliomyelitis vaccine
GP: General practitioner
MMR: Measles, mumps and rubella vaccine
VAERS: Vaccine adverse event reporting system
WHO: World Health Organization.
